# Supplementary material for: An integer GARCH model for a Poisson process with time-varying zero-inflation
Source: PLoS One. 2023 May 18;18(5):e0285769. doi: 10.1371/journal.pone.0285769 (PMC10194996; doi:10.1371/journal.pone.0285769)
Supplement: S1 Appendix — (DOCX) [file pone.0285769.s001.docx]

# S1 Appendix. Derivation of conditions for zero-inflation probability to lie inside (0, 1)

In this appendix we specify and prove the conditions that need to be satisfied to ensure $\omega_{t}\in\left(0,1\right)$ in the sinusoidally varying zero-inflation case.

Let us define sinusoidal zero-inflation function $\omega_{t}=g\left(\mathbf{V}_{t},\mathbf{\Gamma}\right)\in\left(0,1\right)$as given bellow.

$$\omega_{t}=g\left(\mathbf{V}_{t},\mathbf{\Gamma}\right)=A\sin\left(\frac{2\pi}{s}t\right)+B\cos\left(\frac{2\pi}{s}t\right)+C,$$

where$C=\sqrt{A^{2}+B^{2}}+\delta$and $\left|A\right|\leq\frac{1}{2}-\delta$,$\left|B\right|\leq\frac{1}{2}-\delta$, and $\delta\in\left(0,\frac{1}{2}\right)$is a fixed positive value such that $\sqrt{A^{2}+B^{2}}\leq\frac{1}{2}-\delta$. Here .

We consider two possible cases, one in which $\omega_{t}=g\left(\mathbf{V}_{t},\mathbf{\Gamma}\right)$ is a constant over time and the alternative case where it is not.

**Case 1**: If

Since and $A^{2}+B^{2}=0$ implies $A=B=0.$

Therefore,$C=\delta$where $\delta\in\left(0,\frac{1}{2}\right)$then,

$\omega_{t}=g\left(\mathbf{V}_{t},\mathbf{\Gamma}\right)=C$, is a constant over the time span under consideration.

**Case 2:** If $A^{2}+B^{2}\neq0$then,

Here, and. Thus, for a for a fixed $A,B$and $C$, we have

$$\begin{matrix} \max\left(\omega_{t}\right)=\sqrt{A^{2}+B^{2}}+C=2\sqrt{A^{2}+B^{2}}-\delta\leq1-\delta<1, \\ \min\left(\omega_{t}\right)=-\sqrt{A^{2}+B^{2}}+C=\delta, \text{where }\delta\in\left(0,\frac{1}{2}\right). \end{matrix}$$

This implies that the zero-inflation function lines within the interval (0, 1).
