# Supplementary material for: An integer GARCH model for a Poisson process with time-varying zero-inflation
Source: PLoS One. 2023 May 18;18(5):e0285769. doi: 10.1371/journal.pone.0285769 (PMC10194996; doi:10.1371/journal.pone.0285769)
Supplement: S2 Appendix — (DOCX) [file pone.0285769.s002.docx]

## S2 Appendix. Derivation of the conditional mass function

Expression for the conditional mass function for the is derived in this section.

The conditional probability mass function of $Z_{t}\left|\omega_{t}\right.\sim\text{Bernoulli}\left(\omega_{t}\right)$is given by,

$$P\left(Z_{t}=z_{t}\left|\omega_{t}\right.\right)=\omega_{t}^{z_{t}}\left(1-\omega_{t}\right)^{1-z_{t}}.$$

The conditional probability mass function ofis given by,

Therefore, the conditional likelihood function of is: $L\left(\mathbf{\Phi}\right)$=

Therefore, the conditional likelihood function is:

$$l\left(\mathbf{\Phi}\right)=\log(L(\mathbf{\Phi}))=\sum_{t=p+1}^{N} \left\{Z_{t}\log\left(\omega_{t}\right)+\left(1-Z_{t}\right)\left[\log\left(1-\omega_{t}\right)+X_{t}\log\left(\lambda_{t}\right)-\lambda_{t}-\log\left(X_{t}!\right)\right]\right\}.$$
