# Supplementary material for: An integer GARCH model for a Poisson process with time-varying zero-inflation
Source: PLoS One. 2023 May 18;18(5):e0285769. doi: 10.1371/journal.pone.0285769 (PMC10194996; doi:10.1371/journal.pone.0285769)
Supplement: S3 Appendix — (DOCX) [file pone.0285769.s003.docx]

**S3 Appendix. Initialization of the lambdas**

Following tables (Table 1 and Table 2) compare the results of TVZIP-INGARCH (1,1) model when using two initialization values for the $\widehat{\lambda}_{1}^{(0)}$. Table 1 is based on *Real data example - Use of a deterministic sinusoidal zero-inflation function* while Table 2 is prepared based on the *Real data example - zero-inflation function is driven by exogenous variable*.

**Table 1: Estimated parameters, standard errors (within parentheses), model performance, residual diagnostics, and estimated lambda values of TVZIP-INGARCH (1,1) model under two initialization protocols for the pediatric death counts cause by virus A.**

|  | **Initial value** | |
| --- | --- | --- |
|  | $\widehat{\lambda}_{1}^{(0)}=\widehat{\alpha}_{0}^{(0)}$ | $\widehat{\lambda}_{1}^{(0)}=\overline{x}$ |
| **Panel A: Parameter estimates (standard error) of TVZIP-INGARCH (1,1) model** | | |
| $\widehat{A}$ | -0.2643  (0.0402) | -0.2650  (0.0365) |
| $\widehat{B}$ | 0.2550  (0.0404) | 0.2630  (0.0452) |
| $\widehat{\alpha}_{0}$ | 0.2987  (0.1800) | 0.3128  (0.3272) |
| $\widehat{\alpha}_{1}$ | 0.4739  (0.1001) | 0.4758  (0.1444) |
| $\widehat{\beta}_{1}$ | 0.4682  (0.1231) | 0.4618  (0.1116) |
| **Panel B: Model performance of TVZIP-INGARCH (1,1) model** | | |
| AIC | **408.1727** | 408.4778 |
| BIC | **423.5486** | 423.8536 |
| RMSE | **20.1809** | 20.1907 |
| **Panel C: Standardized Pearson residual analysis of TVZIP-INGARCH (1,1) model** | | |
| Mean | 0.0098 | 0.0067 |
| Variance | 0.8902 | 0.8851 |
| **Panel D: Estimated lambda values of TVZIP-INGARCH (1,1) model** | | |
| $\widehat{\lambda}_{1}$ | 0.2987 | 1.5063 |
| $\widehat{\lambda}_{2}$ | 0.4386 | 1.0085 |
| $\widehat{\lambda}_{3}$ | 0.5041 | 0.7786 |
| $\widehat{\lambda}_{4}$ | 0.5348 | 0.6724 |
| $\widehat{\lambda}_{5}$ | 0.5491 | 0.6234 |

**Table 2: Estimated parameters, standard errors (within parentheses), model performance, residual diagnostics, and estimated lambda values of TVZIP-INGARCH (1,1) model under two initialization protocols for the pediatric death counts cause by virus B.**

|  | **Initial value** | |
| --- | --- | --- |
|  | $\widehat{\lambda}_{1}^{(0)}=\widehat{\alpha}_{0}^{(0)}$ | $\widehat{\lambda}_{1}^{(0)}=\overline{x}$ |
| **Panel A: Parameter estimates (standard error) of TVZIP-INGARCH (1,1) model** | | |
| $\widehat{\delta}_{0}$ | -1.0832  (0.2539) | -1.0387  (0.2476) |
| $\widehat{\delta}_{1}$ | 0.1005  (0.0131) | 0.0991  (0.0129) |
| $\widehat{\alpha}_{0}$ | 0.1797  (0.1909) | 0.1677  (0.2009) |
| $\widehat{\alpha}_{1}$ | 0.4090  (0.0947) | 0.4070  (0.0951) |
| $\widehat{\beta}_{1}$ | 0.2493  (0.0680) | 0.2256  (0.0712) |
| **Panel B: Model performance of TVZIP-INGARCH (1,1) model** | | |
| AIC | **441.1931** | 441.4170 |
| BIC | **457.9048** | 458.1286 |
| RMSE | **18.6524** | 18.7076 |
| **Panel C: Standardized Pearson residual analysis of TVZIP-INGARCH (1,1) model** | | |
| Mean-PSR | 0.4857 | 0.5066 |
| Var-PSR | 2.2109 | 2.2935 |
| **Panel D: Estimated lambda values of TVZIP-INGARCH (1,1) model** | | |
| $\widehat{\lambda}_{1}$ | 0.1797 | 0.8517 |
| $\widehat{\lambda}_{2}$ | 0.2245 | 0.3854 |
| $\widehat{\lambda}_{3}$ | 0.2357 | 0.2664 |
| $\widehat{\lambda}_{4}$ | 0.2385 | 0.2357 |
| $\widehat{\lambda}_{5}$ | 0.2392 | 0.2279 |

Results from the tables show that both lambda initialization methods provide the quite similar estimates for the model parameters. However, when considering the model fitting performance, lower values for AIC, BIC and Root Mean Square Error (RMSE) were observed with $\widehat{\lambda}_{1}^{(0)}=\widehat{\alpha}_{0}^{(0)}$. Therefore, in this study we shall proceed with $\widehat{\lambda}_{1}^{(0)}=\widehat{\alpha}_{0}^{(0)}$ initialization method.
