# Supplementary material for: An integer GARCH model for a Poisson process with time-varying zero-inflation
Source: PLoS One. 2023 May 18;18(5):e0285769. doi: 10.1371/journal.pone.0285769 (PMC10194996; doi:10.1371/journal.pone.0285769)
Supplement: S4 Appendix — (DOCX) [file pone.0285769.s004.docx]

# S4 Appendix. List of existing INGARCH type models used in the comparison study

The proposed TVZIP-INGARCH (*p*, *q*) model’s performance was compared with the existing and modified constant zero-inflated integer-valued GARCH models. These models are described as given bellow:

**Table 1: Model Acronym, descriptions, and remarks for the INGARCH models mentioned in the study.**

| **Model Acronym** | **Model Description** | **Remarks** |
| --- | --- | --- |
| **C1: Zero-inflated INGARCH** | | |
| ZIP-INARCH (*p*)  or  ZIP-INGARCH (*p*, *q*) | Zero-inflated Poisson INARCH model with order *p* or  Zero-inflated Poisson INGARCH model with order *p* and *q* | Introduced by Zhu [2] |
| ZINB1-INARCH (*p*)  or  ZINB1-INGARCH (*p*, *q*) | Zero-inflated negative binomial Type 1 INARCH model with order *p* or  Zero-inflated negative binomial Type 1 INGARCH model with order *p* and *q* |
| ZINB2-INARCH (*p*)  or  ZINB2-INGARCH (*p*, *q*) | Zero-inflated negative binomial Type 2 INARCH model with order *p* or  Zero-inflated negative binomial Type 2 INGARCH model with order *p* and *q* |
| ZIGP-INARCH (*p*)  or  ZIGP-INGARCH (*p*, *q*) | Zero-inflated generalized Poisson INARCH model with order *p* or  Zero-inflated generalized Poisson INGARCH model with order *p* and *q* | Introduced by Chen and Lee [18] |
| **C2: Zero-inflated compound Poisson INGARCH** | | |
| ZIGEOMP-INARCH (*p*)  or  ZIGEOMP-INGARCH (*p*, *q*) | Zero-inflated geometric Poisson INARCH model with order *p* or  Zero-inflated geometric Poisson INGARCH model with order *p* and *q* | Introduced by Gonçalves et al. [21] |
| ZINTA-INARCH (*p*)  or  ZINTA-INGARCH (*p*, *q*) | Zero-inflated Neyman Type A INARCH model with order *p* or  Zero-inflated Neyman Type A INGARCH model with order *p* and *q* |
| **C3: Zero-inflated log-linear INGARCH** | | |
| ZIP log-linear INARCH (*p*)  or  ZIP log-linear INGARCH (*p*, *q*) | Zero-inflated Poisson log-linear INARCH model with order *p* or  Zero-inflated Poisson log-linear INGARCH model with order *p* and *q* | We implemented a zero-inflated version of the log-linear INGARCH model of Fokianos and Tjøstheim [10]. In addition to the zero-inflated Poisson, we also used other zero-inflated distributions such as ZINB1, ZINB2, and ZIGP in this implementation.  This model, however, is nested within the general class of models introduced by Xu et al. [30]. |
| ZINB1 log-linear INARCH (*p*)  or  ZINB1 log-linear INGARCH (*p*, *q*) | Zero-inflated negative binomial Type 1 log-linear INARCH model with order *p* or  Zero-inflated negative binomial Type 1 log-linear INGARCH model with order *p* and *q* |
| ZINB2 log-linear INARCH (*p*)  or  ZINB2 log-linear INGARCH (*p*, *q*) | Zero-inflated negative binomial Type 2 log-linear INARCH model with order *p* or  Zero-inflated negative binomial Type 2 log-linear INGARCH model with order *p* and *q* |
| ZIGP log-linear INARCH (*p*)  or  ZIGP log-linear INGARCH (*p*, *q*) | Zero-inflated generalized Poisson log-linear INARCH model with order *p* or  Zero-inflated generalized Poisson log-linear INGARCH model with order *p* and *q* |
| **C4: Zero-inflated log-linear INGARCHX** | | |
| ZIP log-linear INARCHX (*p*)  or  ZIP log-linear INGARCHX (*p*, *q*) | Zero-inflated Poisson log-linear INARCHX model with order *p or*  Zero-inflated Poisson log-linear INGARCHX model with order *p* and *q* | We implemented zero-inflated versions of the log-linear INGARCHX model of Chen and Lee [11] with the underlying distributions of ZIP, ZINB1, ZINB2, and ZIGP in this implementation.  This model, however, is nested within the general class of models introduced by Xu et al. [30]. |
| ZINB1 log-linear INARCHX (*p*)  or  ZINB1 log-linear INGARCHX (*p*, *q*) | Zero-inflated negative binomial Type 1 log-linear INARCHX model with order *p* or  Zero-inflated negative binomial Type 1 log-linear INGARCHX model with order *p* and *q* |
| ZINB2 log-linear INARCHX (*p*)  or  ZINB2 log-linear INGARCHX (*p*, *q*) | Zero-inflated negative binomial Type 2 log-linear INARCHX model with order *p* or  Zero-inflated negative binomial Type 2 log-linear INGARCHX model with order *p* and *q* |
| ZIGP log-linear INGARCHX (*p*)  or  ZIGP log-linear INGARCHX (*p*, *q*) | Zero-inflated generalized Poisson log-linear INARCHX model with order *p* or  Zero-inflated generalized Poisson log-linear INGARCHX model with order *p* and *q* |
| **C5: Zero-inflated softplus INGARCH** | | |
| ZIP softplus INGARCH (*p*)  or  ZIP softplus INGARCH (*p*, *q*) | Zero-inflated Poisson softplus INARCH model with order *p*  or Zero-inflated Poisson softplus INGARCH model with order *p* and *q* | We generalized the softplus INGARCH model of Weiß et al. [12] to include zero-inflation. In addition to the zero-inflated Poisson we included other zero-inflated distributions such as ZINB1, ZINB2, and ZIGP. |
| ZINB1 softplus INGARCH (*p*)  or  ZINB1 softplus INGARCH (*p*, *q*) | Zero-inflated negative binomial Type 1 softplus INARCH model with order *p* or  Zero-inflated negative binomial Type 1 softplus INGARCH model with order *p* and *q* |
| ZINB2 softplus INGARCH (*p*)  or  ZINB2 softplus INGARCH (*p*, *q*) | Zero-inflated negative binomial Type 2 softplus INARCH model with order *p* or  Zero-inflated negative binomial Type 2 softplus INGARCH model with order *p* and *q* |
| ZIGP softplus INGARCH (*p*)  or  ZIGP softplus INGARCH (*p*, *q*) | Zero-inflated generalized Poisson softplus INARCH model with order *p* or  Zero-inflated generalized Poisson softplus INGARCH model with order *p* and *q* |
| **C6: Zero-inflated softplus INGARCHX** | | |
| ZIP softplus INARCHX (*p*)  or  ZIP softplus INGARCHX (*p*, *q*) | Zero-inflated Poisson softplus INARCHX model with order *p*  or  Zero-inflated Poisson softplus INGARCHX model with order *p* and *q* | We extended the softplus INGARCH model of Weiß et al. [12] to accommodate an exogenous variable and extended the zero-inflated distributions to include ZINB1, ZINB2, and ZIGP in addition to ZIP. |
| ZINB1 softplus INARCHX (*p*)  or  ZINB1 softplus INGARCHX (*p*, *q*) | Zero-inflated negative binomial Type 1 softplus INARCHX model with order *p*  or  Zero-inflated negative binomial Type 1 softplus INGARCHX model with order *p* and *q* |
| ZINB2 softplus INARCHX (*p*)  or  ZINB2 softplus INGARCHX (*p*, *q*) | Zero-inflated negative binomial Type 2 softplus INARCHX model with order *p*  or  Zero-inflated negative binomial Type 2 softplus INGARCHX model with order *p* and *q* |
| ZIGP softplus INARCHX (*p*)  or  ZIGP softplus INGARCHX (*p*, *q*) | Zero-inflated generalized Poisson softplus INARCHX model with order *p*  or  Zero-inflated generalized Poisson softplus INGARCHX model with order *p* and *q* |

Note: (1) The models in cells in light grey are generalizations we introduced to existing models.

(2) In the above INGARCH model of *p* and *q*, if $p>0$and$q=0,$then the model reduces to an INARCH model with order *p*.
