# Supplementary material for: An integer GARCH model for a Poisson process with time-varying zero-inflation
Source: PLoS One. 2023 May 18;18(5):e0285769. doi: 10.1371/journal.pone.0285769 (PMC10194996; doi:10.1371/journal.pone.0285769)
Supplement: S5 Appendix — (DOCX) [file pone.0285769.s005.docx]

# S5 Appendix. Model comparison results for the pediatric death counts cause by virus A

In this appendix we present the model comparison results for the TVZIP-INGARCH model with the other constant zero-inflated probability versions of INGARCH, compound Poisson INGARCH, log-linear INGARCH, and softplus INGARCH models. The AIC and BIC values form the model fit are listed in Table 1A (model category C0 with scenario Sc1 and Sc2) and Table 1B (model category C1, C2, C3, C5 with scenario Sc3). The summary statistics for the standardized Pearson residuals are given in Table 2.

**Table 1A: Model fitting for pediatric death counts caused by virus A: The AIC and BIC values for versions of the proposed model (Model category C0, with model order M1, M2, M3 and scenario Sc1 and Sc2).**

| **Zero-inflation scenario** | **Model**  **order** | **Model category** | **Model selection criteria** | |
| --- | --- | --- | --- | --- |
|  | | **C0: Time-varying zero-inflated Poisson INGARCH** | **AIC** | **BIC** |
| **Sc1** | M1 | TVZIP-INARCH (1) | 411.8966 | 424.1973 |
|  | M2 | ***TVZIP-INARCH (2)*** | ***398.7016*** | ***414.0774*** |
|  | M3 | TVZIP-INGARCH (1,1) | 402.5728 | 417.9487 |
| **Sc2** | M1 | TVZIP-INARCH (1) | 419.6800 | 431.9807 |
|  | M2 | ***TVZIP-INARCH (2)*** | ***403.6973*** | ***419.0732*** |
|  | M3 | TVZIP-INGARCH (1,1) | 408.1727 | 423.5486 |

**Table 1B: Model fitting for pediatric death counts caused by virus A: The AIC and BIC values for models in categories C1, C2, C3, C5 with model orders M1, M2, M3 and scenario Sc3.**

| **Zero-inflation Scenario** | **Model**  **Order** | **Model Category** | **Model selection criteria** | |
| --- | --- | --- | --- | --- |
|  |  | **C1: Zero-inflated INGARCH** | **AIC** | **BIC** |
| **Sc3** | M1 | ZIP-INARCH (1) | 451.6289 | 460.8544 |
|  |  | ZINB1-INARCH (1) | 435.5160 | 447.7916 |
|  |  | ZINB2-INARCH (1) | 439.4161 | 451.6917 |
|  |  | ZIGP-INARCH (1) | 435.7900 | 448.0656 |
|  |  | ZINB1-INARCH (1) - change point | 430.4803 | 449.3426 |
|  | M2 | ZIP-INARCH (2) | 419.8733 | 432.1740 |
|  |  | **ZINB1-INARCH (2)** | **412.3557** | **427.6686** |
|  |  | ZINB2-INARCH (2) | 415.3867 | 430.6997 |
|  |  | ZIGP-INARCH (2) | 412.4033 | 427.7163 |
|  |  | **ZINB1-INARCH (2) - change point** | **410.2882** | **433.6915** |
|  | M3 | ZIP-INGARCH (1,1) | 420.6112 | 432.9119 |
|  |  | ZINB1-INGARCH (1,1) | 473.6701 | 489.0146 |
|  |  | ZINB2-INGARCH (1,1) | 464.6901 | 480.0347 |
|  |  | ZIGP-INGARCH (1,1) | 422.8282 | 438.1412 |
|  |  | ZIP-INGARCH (1,1) - change point | 418.2907 | 437.2558 |
|  |  | **C2: Zero-inflated compound Poisson INGARCH** | **AIC** | **BIC** |
| **Sc3** | M1 | ZIGEOMP-INARCH (1) | 433.4213 | 447.6969 |
|  |  | ZINTA-INARCH (1) | 436.1792 | 448.4548 |
|  | M2 | ***ZIGEOMP-INARCH (2)*** | ***412.4334*** | ***427.7463*** |
|  |  | ZINTA-INARCH (2) | 413.5864 | 428.8994 |
|  | M3 | ZIGEOMP-INGARCH (1,1) | 415.3122 | 430.6567 |
|  |  | ZINTA-INGARCH (1,1) | 415.8081 | 431.1526 |
|  |  | **C3: Zero-inflated log-linear INGARCH** | **AIC** | **BIC** |
| **Sc3** | M1 | ZIP log-linear INARCH (1) | 457.3196 | 469.5952 |
|  |  | ZINB1 log-linear INARCH (1) | 437.2397 | 449.5153 |
|  |  | ZINB2 log-linear INARCH (1) | 446.7800 | 459.0557 |
|  |  | ZIGP log-linear INARCH (1) | 441.2088 | 453.4845 |
|  | M2 | ZIP log-linear INARCH (2) | 433.0241 | 448.3370 |
|  |  | ***ZINB1 log-linear INARCH (2)*** | ***418.3061*** | ***433.6191*** |
|  |  | ZINB2 log-linear INARCH (2) | 422.3263 | 437.6393 |
|  |  | ZIGP log-linear INARCH (2) | 418.4273 | 433.7403 |
|  | M3 | ZIP log-linear INGARCH (1,1) | 441.2279 | 453.5035 |
|  |  | ZINB1 log-linear INGARCH (1,1) | 458.2238 | 470.4994 |
|  |  | ZINB2 log-linear INGARCH (1,1) | 426.0062 | 438.2818 |
|  |  | ZIGP log-linear INGARCH (1,1) | 424.1644 | 436.4400 |
|  |  | **C5: Zero-inflated softplus INGARCH** | **AIC** | **BIC** |
| **Sc3** | M1 | ZIP softplus INARCH (1) | 461.1621 | 473.4377 |
|  |  | ZINB1 softplus INARCH (1) | 438.5905 | 450.8661 |
|  |  | ZINB2 softplus INARCH (1) | 443.3884 | 455.6640 |
|  |  | ZIGP softplus INARCH (1) | 440.9086 | 453.1842 |
|  | M2 | ZIP softplus INARCH (2) | 430.5373 | 445.8502 |
|  |  | ***ZINB1 softplus INARCH (2)*** | ***416.8903*** | ***432.2032*** |
|  |  | ZINB2 softplus INARCH (2) | 420.0888 | 435.4018 |
|  |  | ZIGP softplus INARCH (2) | 419.0470 | 434.3600 |
|  | M3 | ZIP softplus INGARCH (1,1) | 442.1361 | 454.4117 |
|  |  | ZINB1 softplus INGARCH (1,1) | 442.9594 | 455.2350 |
|  |  | ZINB2 softplus INGARCH (1,1) | 438.9889 | 451.2645 |
|  |  | ZIGP softplus INGARCH (1,1) | 467.7912 | 480.0668 |

Note: (a). Within each model category by model order combination the best model with the lowest AIC is highlighted in light gray; (b). When BIC was used instead of AIC, the same models were selected; (c) For models that fall into the zero-inflated INGARCH category only, the residual based CUSUM test of Lee et al. [27] was performed to detect a possible change point; (d). If a change point was detected using the residuals from the selected model, then the same model was fitted to the sub-series before and after the change point; (e). The AIC and BIC values for this change point model are also included in the list of models above; (f). For each model category, the model with the lowest information criteria value according to both AIC and BIC from among those highlighted in light gray are indicated in italic boldface font; (g). For each model category, the model with the lowest information criteria values according to one of AIC or BIC but not both are indicated in boldface font.

**Table 2. Summary statistics for standard Pearson residuals analysis for TVZIP-INARCH (2) model.**

| **Scenario** | **Model structure** | **Mean of the Fitted value** | **Standardized Pearson Residuals** | |
| --- | --- | --- | --- | --- |
|  |  |  | **mean** | **variance** |
| Sc1 | TVZIP-INARCH (2) | 1.4608 | 0.0117 | 0.8108 |
