# Supplementary material for: An integer GARCH model for a Poisson process with time-varying zero-inflation
Source: PLoS One. 2023 May 18;18(5):e0285769. doi: 10.1371/journal.pone.0285769 (PMC10194996; doi:10.1371/journal.pone.0285769)
Supplement: Attachment — Submitted filename: Response to Reviewers.pdf [file pone.0285769.s010.pdf]

Isuru Ratnayake,

4/13/2023,

Cathy W.S. Chen, Ph.D.,

Academic Editor,

PLOS ONE.

Dear Dr. Chen,

Thank you for the invitation to submit a revised version of our manuscript (PONE-D-22-26550R1). We wish to thank you and the reviewers for their valuable time and insightful comments. These have greatly helped us to improve the quality of our manuscript. We considered the points raised by the academic editor during the review process, and carefully addressed them as described below.

> We updated the abstract and the new version of the abstract is self-contained and entirely understandable without reference to other sources.

We believe that this new version of the manuscript incorporates all main aspects pointed out by the editor and hope that it satisfies the journal requirements and meets the high standards for publication in PLOS ONE.

I am looking forward to hearing from you at your earliest convenience.

Thank you,

Your Sincerely,

……………………………………

Isuru Ratnayake.
