# Supplementary material for: An integer GARCH model for a Poisson process with time-varying zero-inflation
Source: PLoS One. 2023 May 18;18(5):e0285769. doi: 10.1371/journal.pone.0285769 (PMC10194996; doi:10.1371/journal.pone.0285769)
Supplement: Attachment — Submitted filename: Response to Reviewers .docx [file pone.0285769.s011.docx]

Isuru Ratnayake,

4/21/2023,

Cathy W.S. Chen, Ph.D.,

Academic Editor,

PLOS ONE.

Dear Dr. Chen,

Thank you for inviting us to submit a revised version of our manuscript (PONE-D-22-26550R2). We greatly appreciate the time and insightful comments from you and the reviewers, which have helped us improve the quality of our manuscript.

We have carefully considered the points raised by the academic editor during the review process and addressed them accordingly. In particular, we have updated the abstract to be self-contained and entirely understandable without reference to other sources.

We believe that this new version of the manuscript incorporates all the main aspects pointed out by the editor and hope that it satisfies the journal’s requirements and meets its high standards for publication in PLOS ONE.

In addition to this, we would like to mention that we contacted you to respectfully explain the revised abstract. You replied that the paper could be accepted (Author query to editor-PONE-D-22-26550R2, case Number: 07982474). However, we are submitting it again because the Editorial Manager web page has stated that the submission still needs revision from the author.

We are truly grateful for your time and apologize if we have created any additional burden.

Thank you,

Your Sincerely,

…………………………………

Isuru Ratnayake.

The revised abstract is provided below:

**Abstract**

A serially dependent Poisson process with time-varying zero-inflation is proposed. Such formulations have the potential to model count data time series arising from phenomena such as infectious diseases that ebb and flow over time. The model assumes that the intensity of the Poisson process evolves according to a generalized autoregressive conditional heteroscedastic (GARCH) formulation and allows the zero-inflation parameter to vary over time and be governed by a deterministic function or by an exogenous variable. Both the expectation maximization (EM) and the maximum likelihood estimation (MLE) approaches are presented as possible estimation methods. A simulation study shows that both parameter estimation methods provide good estimates. Applications to two real-life data sets on infant deaths due to influenza show that the proposed integer-valued GARCH (INGARCH) model provides a better fit in general than existing zero-inflated INGARCH models. We also extended a non-linear INGARCH model to include zero-inflation and an exogenous input. This extended model performed as well as our proposed model with respect to some criteria, but not with respect to all.
